# Editorial: 15 years of Frontiers in Human Neuroscience: risk factors and prevention in common disorders of the brain

**DOI:** 10.3389/fnhum.2024.1454187

**Published:** 2024-07-09

**Authors:** Virginie Lam, Daniele Corbo

**Affiliations:** ^1^School of Population Health, Curtin Health Innovation Research Institute, Faculty of Health Sciences, Curtin University, Perth, WA, Australia; ^2^Curtin Health Innovation Research Institute, Faculty of Health Sciences, Curtin University, Perth, WA, Australia; ^3^Department of Medical Surgical Specialties, Radiological Sciences and Public Health, University of Brescia, Brescia, Italy

**Keywords:** risk factors, neuroimaging, brain disorder, prevention, cognitive impairment

Numerous scientific data point to the shared underlying cellular and molecular mechanisms of neurodegenerative disorders and neuropsychiatric disorders (such as anxiety, major depressive disorder, bipolar affective disorder, and schizophrenia) in terms of oxidative stress and neuroinflammation. Primary care medicine has long prioritized the prevention of these disorders of the central nervous system (CNS). It is now understood that modifiable risk factors account for up to 40% of dementia, stroke, and depression cases. Thus, identifying the potentially modifiable causes for CNS illnesses will aid in the implementation of efficient long-term brain health improvement strategies. Variations in an individual's surroundings account for around 50% of the risk of complicated diseases. Obesity, hypertension, diabetes, and smoking are just a few of the lifestyle choices and cardiovascular risk factors that have been shown to increase the incidence of CNS illnesses. Studies have indicated that communities experiencing overall levels of social and economic inequality are more likely to experience stress-related diseases, such as anxiety and depression. Maternal infection is a significant risk factor for developing psychiatric problems, according to epidemiological studies. Inflammation, oxidative stress, and cerebrovascular abnormalities are a few other pathophysiological processes that contribute to the etiology of Alzheimer's disease (AD). Poor dental health has been recognized as a risk factor for the onset of AD relatively recently. There is evidence that oral health conditions, especially periodontitis, increase the risk of exacerbating neuroinflammation, which can lead to chronically elevated pro-inflammatory status, among other consequences. Air pollution has been linked to neurodevelopmental delays, attention deficit disorders, autism, and ADHD in children and adolescents, as well as cognitive decline and an increased risk of AD, Parkinson's disease, amyotrophic lateral sclerosis, and related disorders in late adulthood. ADHD cannot be caused by a single risk factor, either essential or sufficient. The heterogeneity of ADHD reflects its multifactorial causation, as evidenced by the wide range of structural and functional brain anomalies, psychiatric comorbidities, clinical profiles, neurocognitive impairment patterns, and developmental trajectories. Luo et al. carried out a thorough assessment of the state of research on the heterogeneity of ADHD. They recommend concentrating future research efforts on examining the effects of the etiological risk factors and how they interact with brain pathways involved in development and clinical characteristics of ADHD. A syndrome known as isolated rapid eye movement behavior disorder (iRBD), in which over 80% of patients later develop Parkinson's disease, dementia with Lewy bodies, or multiple system atrophy, may be a possible preclinic indication of neurodegenerative synucleinopathies. Finding risk factors is therefore essential for screening high-risk groups and lowering the prevalence of iRBD, especially those that can be addressed. More insights and evidence for screening and early intervention in at-risk groups were provided by Zhang et al.'s mendelian randomization technique to assessing the causality between these variables and iRBD. Migraines, a widespread condition that often starts in infancy, are most common throughout adolescence and the early stages of adulthood. Colon et al. planned to compare age-matched healthy controls to migraineurs in terms of resting state functional connectivity at two developmental phases. They aimed to understand how significant neurodevelopmental changes occur from adolescence to adulthood affect brain function and suggest that the condition may respond differently to certain treatments. Conditions that cause injury or dysfunction in the parts of the CNS that regulate arousal and awareness are referred to as disorders of consciousness. A clinical prediction model based on these characteristics was established and verified by Liu et al. with the goal of investigating the electroencephalogram indicators and clinical factors that may contribute to a poor prognosis in patients with protracted disturbance of consciousness. This model provides medical professionals with an impartial and reliable instrument to assess prognosis of prolonged disorder of consciousness, and they support them in developing individualized therapeutic decisions and care plans for patients and their families. Preoperative cognitive impairment (PCI) may increase the likelihood of postoperative delirium (POD), however, however PCI screening is not frequently done. POD lengthens hospital stays, raises medical expenses, and raises mortality and morbidity rates in senior citizens. Li et al. showed the Mini-Cog test for preoperative cognitive screening is a quick and valid cognitive assessment that requires significantly less time than other scales. By screening for PCI, elderly thoracic patients may receive individualized therapy that helps to reduce the risk of unfavorable postoperative outcomes. Although there are many arguments on this Research Topic contributing to the scientific discussion about risk factors that might lead to brain disorders, it is undoubtedly just the beginning of a deeper and more comprehensive examination of the subject. Disease-modifying and risk variables would attract a great lot of attention because of the substantial socioeconomic burden associated with brain disorders, which are an emerging public health concern. Despite the good work that has been done, there is still much to understand about the numerous approaches used to avoid these disorders and their etiopathogenesis.

## Author contributions

DC: Writing – original draft. VL: Writing – review & editing.

